# Accuracy of P0.1 measurements performed by ICU ventilators: a bench study

**DOI:** 10.1186/s13613-019-0576-x

**Published:** 2019-09-13

**Authors:** François Beloncle, Lise Piquilloud, Pierre-Yves Olivier, Alice Vuillermoz, Elise Yvin, Alain Mercat, Jean-Christophe Richard

**Affiliations:** 1 Medical Intensive Care Unit, University Hospital of Angers, UNIV Angers, 4 rue Larrey, 49933 Angers Cedex 9, France; 2 Adult Intensive Care and Burn Unit, University Hospital and University of Lausanne, Lausanne, Switzerland; 3 Intensive Care Unit, General Hospital of Le Mans, Le Mans, France; 4 SAMU74, Emergency Department, General Hospital of Annecy, Annecy, France; 5 INSERM, UMR 1066, Creteil, France

**Keywords:** Mechanical ventilation, Occlusion pressure, Respiratory drive, Inspiratory effort, Respiratory failure

## Abstract

**Background:**

Occlusion pressure at 100 ms (P0.1), defined as the negative pressure measured 100 ms after the initiation of an inspiratory effort performed against a closed respiratory circuit, has been shown to be well correlated with central respiratory drive and respiratory effort. Automated P0.1 measurement is available on modern ventilators. However, the reliability of this measurement has never been studied. This bench study aimed at assessing the accuracy of P0.1 measurements automatically performed by different ICU ventilators.

**Methods:**

Five ventilators set in pressure support mode were tested using a two-chamber test lung model simulating spontaneous breathing. P0.1 automatically displayed on the ventilator screen (P0.1_vent_) was recorded at three levels of simulated inspiratory effort corresponding to P0.1 of 2.5, 5 and 10 cm H_2_O measured directly at the test lung and considered as the reference values of P0.1 (P0.1_ref_). The pressure drop after 100 ms was measured offline on the airway pressure–time curves recorded during the automated P0.1 measurements (P0.1_aw_). P0.1_vent_ was compared to P0.1_ref_ and to P0.1_aw_. To assess the potential impact of the circuit length, P0.1 were also measured with circuits of different lengths (P0.1_circuit_).

**Results:**

Variations of P0.1_vent_ correlated well with variations of P0.1_ref_. Overall, P0.1_vent_ underestimated P0.1_ref_ except for the Löwenstein^®^ ventilator at P0.1_ref_ 2.5 cm H_2_O and for the Getinge group^®^ ventilator at P0.1_ref_ 10 cm H_2_O. The agreement between P0.1_vent_ and P0.1_ref_ assessed with the Bland–Altman method gave a mean bias of − 1.3 cm H_2_O (limits of agreement: 1 and − 3.7 cm H_2_O). Analysis of airway pressure–time and flow–time curves showed that all the tested ventilators except the Getinge group^®^ ventilator performed an occlusion of at least 100 ms to measure P0.1. The agreement between P0.1_vent_ and P0.1_aw_ assessed with the Bland–Altman method gave a mean bias of 0.5 cm H_2_O (limits of agreement: 2.4 and − 1.4 cm H_2_O). The circuit’s length impacted P0.1 measurements’ values. A longer circuit was associated with lower P0.1_circuit_ values.

**Conclusion:**

P0.1_vent_ relative changes are well correlated to P0.1_ref_ changes in all the tested ventilators. Accuracy of absolute values of P0.1_vent_ varies according to the ventilator model. Overall, P0.1_vent_ underestimates P0.1_ref_. The length of the circuit may partially explain P0.1_vent_ underestimation.

## Introduction

There is a growing interest in better understanding beneficial and/or potentially harmful effects associated with spontaneous breathing in mechanical ventilation. The concept of patient self-inflicted lung injury (P-SILI) that recently emerged from the literature, teaches us that assisted ventilation can be injurious when respiratory drive is high [1, 2]. During assisted ventilation, high respiratory drive is associated with strong inspiratory efforts and very negative pleural pressure resulting in major stress applied to the lung parenchyma [1]. Furthermore, both too low and too high respiratory drive are recognized as risk factors for diaphragmatic injury [3]. In this context, the use of airway occlusion pressure at 100 ms (P0.1) which is usually considered as the simplest way to assess respiratory drive at the bedside, is of major interest for ventilated patients’ management [4, 5]. P0.1 is defined as the negative pressure measured at the airway opening 100 ms after the initiation of an inspiratory effort performed against a closed respiratory circuit [6–8]. P0.1 measurement is not perceived by the patient and does not influence respiratory pattern. More importantly, since P0.1 measurement is performed during an occlusion at the onset of the breath, flow and insufflated volume are equal to zero at the time of measurement and P0.1 is unrelated to respiratory mechanics. P0.1 measurement is feasible and reliable in presence of inspiratory muscle weakness [5–7], abnormal respiratory compliance [6–8] or intrinsic positive end expiratory pressure [8–10]. P0.1 measurement was initially described in non-intubated spontaneously breathing patients. In these patients, it has been shown to be correlated with central respiratory drive and respiratory effort [11]. In spontaneously breathing non-ventilated healthy subjects, low values around 2 cm H_2_O are observed with normal respiratory drive whereas values greater than 10 cm H_2_O are correlated with very high drive [6]. P0.1 measurement was later used in intubated patient by occluding the ventilator circuit [12, 13]. Automated measurement is now available in modern ICU ventilators either by default or on request using an easy-to-use and safe maneuver [14]. However, the reliability of P0.1 automated measurements displayed by the different ICU ventilators has never systematically been studied. This study aimed at assessing the accuracy of ventilator automated P0.1 measurements. Using a lung model of an invasively ventilated spontaneously breathing patient, we compared the P0.1 displayed by several ventilators (P0.1_vent_) to reference values of P0.1 (P0.1_ref_) simulated on the lung model.

## Materials and methods

### Test lung and calibration of respiratory efforts

To simulate spontaneous ventilation, a two-chamber Michigan test lung (Michigan Instruments, Grand Rapids, USA) was connected to a driving ventilator set in volume-controlled ventilation mode with constant inspiratory flow (Dräger, Evita 4^®^, Lübeck, Germany) at a respiratory rate of 8/min as previously described [15]. Reference P0.1 (P0.1_ref_) were obtained by occluding the second compartment of the test lung at the airway opening using an hermetic plug. The tidal volumes and inspiratory times of the driving ventilator were set to obtain P0.1_ref_ of 2.5, 5 and 10 cm H_2_O corresponding to low, moderate and strong respiratory efforts, respectively. The compliance and resistance of the two chambers were set to 60 mL/cm H_2_O and 5 cm H_2_O/L/s, respectively. To transmit the pressure generated by the driving ventilator to the tested ventilator, the two chambers of the test lung were linked by a rigid metal piece. Thus, the positive pressure insufflated by the driving ventilator into the first chamber generated a negative pressure in the second chamber. This negative pressure was recognized as an inspiratory effort by the tested ventilator.

**Fig. 1:**
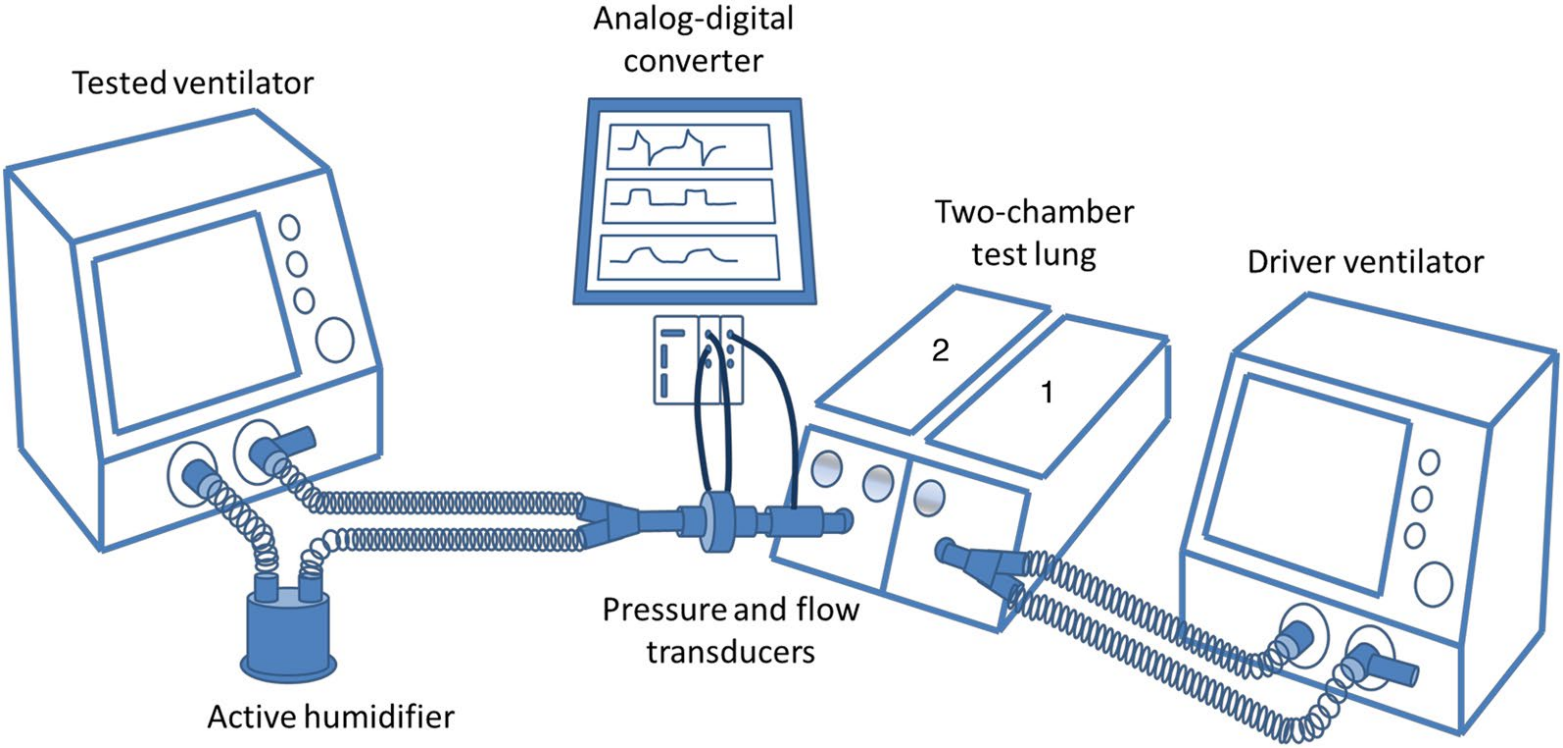
Experimental setup. A chamber of a two-chamber test lung was connected to a driving ventilator set in volume-controlled ventilation mode. The second chamber (2) was connected to the tested ventilator by a double limb circuit with an active humidifier. The two chambers of the test lung were linked by a rigid metal piece, so that the positive pressure insufflated by the driving ventilator into the first chamber (1) generates a negative pressure in the second chamber, recognized as an inspiratory effort by the tested ventilator. Data were acquired via an analog-to-digital converter and stored in a laptop computer for subsequent analysis

The tested ventilator was connected to the second chamber through a double limb circuit with an active humidifier (total length of circuit tubes of 360 cm including the inspiratory and expiratory limbs) (Fig. 1) for baseline measurements.

To investigate the potential impact of the circuit tubes’ length on P0.1 measurements, an occlusion of the second compartment of the test lung was performed using a hermetic plug positioned at the airway opening and at the end of circuit tubes of different lengths, at 195, 360 or 690 cm away from the test lung second chamber. P0.1 values (referenced as P0.1_circuit_) corresponding to each simulated effort (P0.1_ref_ of 2.5, 5 and 10 cm H_2_O, respectively) were measured with the different occlusion locations.

To record pressure– and flow–time curves, pressure and flow transducers (pneumotachograph, Biopac Systems^®^, Goleta, CA, USA) were inserted at the airway opening. More specifically, the transducers were placed between the test lung second chamber and the Y piece of the ventilator circuit for P0.1_aw_ measurement. They were placed between the second chamber of the test lung and the hermetic plug for P0.1_ref_ measurements. For P0.1_circuit_ measurements, the pressure and flow transducers were also inserted at the airway opening (i.e., between the second chamber of the test lung and the circuit). Signals were acquired using an analog-to-digital converter (MP150; Biopac Systems^®^) sampled at 50 Hz. Subsequent offline analysis was performed using a dedicated software (AcqKnowledge software version 4.2, Biopac systems^®^).

All measurements were performed in Ambient Temperature and Pressure Saturated (ATPS) conditions.

### Tested ventilators

Five commercialized ventilators (Covidien, PB 980^®^, Carlsbad, USA; Dräger, Evita 4^®^, Lübeck, Germany; GE Healthcare, Carescape R860^®^, Madison, USA; Löwenstein Medical, Elisa 800^®^, Bad Ems, Germany; Getinge group, Servo-u^®^, Solna, Sweden) were tested in pressure support mode (positive end expiratory pressure of 5 cm H_2_O, pressure support level of 10 cm H_2_O, flow inspiratory trigger of 2 L/min, inspiratory slope of 0 and FiO_2_ of 21%).

Each tested ventilator was assessed for the three levels of inspiratory intensity (corresponding to P0.1_ref_ of 2.5, 5 and 10 cm H_2_O, respectively).

### Recorded, measured and computed parameters

P0.1 automatically displayed on the ventilator screens (P0.1_vent_) were recorded for each inspiratory effort intensity.

Based on recorded airway pressure–time curves, occlusions automatically performed by the ventilators to measure P0.1 were identified. For the ventilators performing an automatic occlusion of more than 100 ms to measure P0.1, P0.1_aw_ was measured on the airway pressure–time curves during an automatically performed occlusion as the drop in pressure between airway pressure at end-expiration and airway pressure 100 ms after the beginning of the inspiratory effort (Additional file 1: Figure S1). For the ventilators performing an occlusion of less than 100 ms, the drop in airway pressure during either the trigger delay time or the dedicated occlusion was used to estimate P0.1_aw_. Practically, the slope of the considered airway pressure drop was calculated and used to extrapolate the amplitude of the pressure drop after 100 ms.

To assess the effect of the circuit length on P0.1 measurements, we computed ∆P0.1_circuit_ defined as the difference between P0.1_ref_ measured at the airway opening and P0.1_circuit_ measured by performing an occlusion at the end of a standard ventilator circuit (360 cm away from the airway opening). P0.1_vent corrected_ was defined as P0.1_vent corrected_ = P0.1_vent_ + ∆P0.1_circuit_.

### Data analysis and statistics

For each condition, the previously described P0.1 values were recorded or measured five times. As the five measurements were very similar, the results are presented as the mean of the five values.

A Bland and Altman plot of differences between P0.1_vent_ and P0.1_ref_ versus their mean was constructed to evaluate agreement between these two values [16]. Similarly, Bland and Altman plot between P0.1_vent_ and P0.1_aw_ was also performed. The statistical analysis was performed using Prism (GraphPad Software, La Jolla, CA, USA).

## Results

### Correlation between P0.1 displayed on the ventilator screen (P0.1_vent_) and P0.1 set on the lung model (P0.1_ref_)

**Fig. 2:**
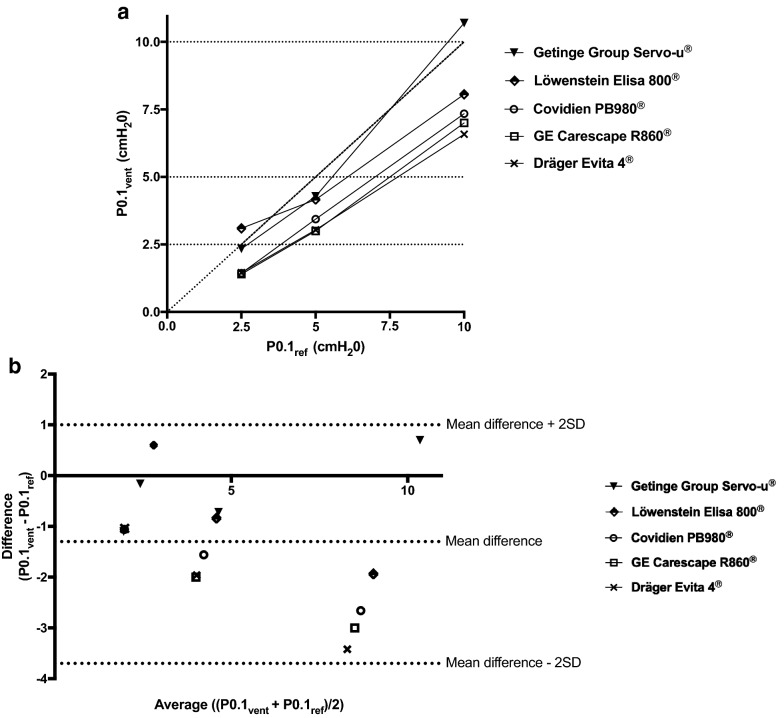
Accuracy of automatically measured P0.1 displayed on the ventilator screen (P0.1_vent_). **a** Correlation between P0.1_vent_ and reference P0.1 (P0.1_ref_). The horizontal dotted lines represent P0.1 reference values of 2.5, 5 and 10 cm H_2_O, respectively. The diagonal dotted line represents the identity line (*y* = *x*). **b** Bland and Altman plots of differences between P0.1_vent_ and P0.1_ref_ versus their mean. The dotted lines represent the mean bias and the limits of agreement

The correlation between P0.1_vent_ and P0.1_ref_ is illustrated in Fig. 2. Variations of P0.1_vent_ correlated well with variations of P0.1_ref_ (Fig. 2a). Overall, P0.1_vent_ underestimated P0.1_ref_ except for the Löwenstein Elisa 800^®^ at P0.1_ref_ 2.5 cm H_2_O and for the Getinge Group Servo-u^®^ at P0.1_ref_ 10 cm H_2_O. The Bland–Altman plot revealed a mean bias of − 1.3 cm H_2_O, with limits of agreement of 1.0 and − 3.7 cm H_2_O (Fig. 2b).

### Automated measurement techniques

Visual analysis of airway pressure–time and flow–time curves showed that all the tested ventilators except the Getinge group Servo-u^®^ ventilator performed an occlusion of more than 100 ms to measure P0.1. Two representative tracings are presented in Additional file 2: Figure S2. Of note, in the ventilators performing an automatic occlusion to measure P0.1, flow was not strictly equal to zero during the occlusion.

### Correlation between P0.1 displayed on the ventilator screen (P0.1_vent_) and P0.1 measured on the airway pressure curve (P0.1_aw_)

**Fig. 3:**
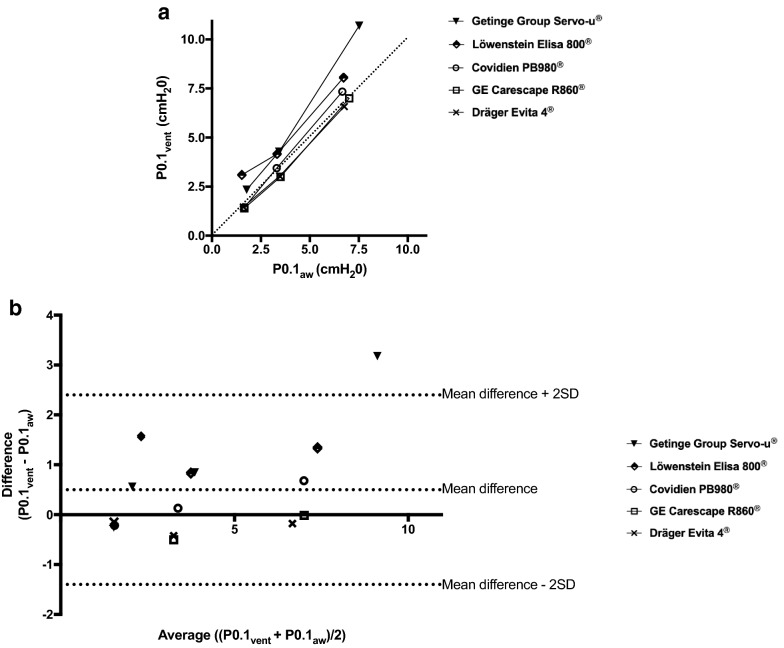
Relationship between automatically measured P0.1 displayed by the ventilator (P0.1_vent_) and P0.1 measured on the airway pressure during automated P0.1 measurements (P0.1_aw_). **a** Correlation between P0.1_vent_ and P0.1_aw_. The dotted line represents the identity line (*y* = *x*). **b** Bland and Altman plots of differences between P0.1_vent_ and P0.1_aw_ versus their mean. The dotted lines represent the mean bias and the limits of agreement

P0.1_aw_ values were well correlated to P0.1_vent_ values (Fig. 3a). The Bland–Altman plot revealed a mean bias of 0.5 cm H_2_O, with limits of agreement of 2.4 and − 1.4 cm H_2_O (Fig. 3b).

### Effect of circuit occlusion position on P0.1 measurements

**Fig. 4:**
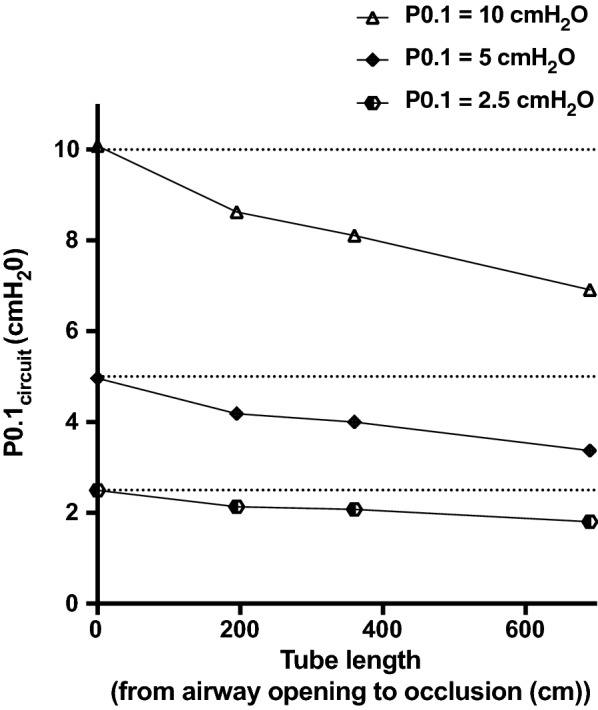
P0.1 measurements (P0.1_circuit_) according to the position of the airway occlusion. P0.1_circuit_ measurements were performed with occlusions at different positions (i.e., at 0, 195, 360 and 690 cm from the airway opening). The second compartment of the test lung was occluding using a hermetic plug placed at the airway opening or on a circuit tube of 195, 360 or 690 cm connected to the airway opening. Dotted lines represent P0.1 values of 2.5, 5 and 10 cm H_2_O, respectively

At the three levels of P0.1_ref_, the location of the circuit occlusion strongly impacted P0.1_circuit_ values (Fig. 4). A longer circuit was associated with lower values of P0.1_circuit_. ∆P0.1_circuit_ was 0.42, 0.97 and 1.97 cm H_2_O for P0.1_ref_ of 2.5, 5 and 10 cm H_2_O, respectively.

**Fig. 5:**
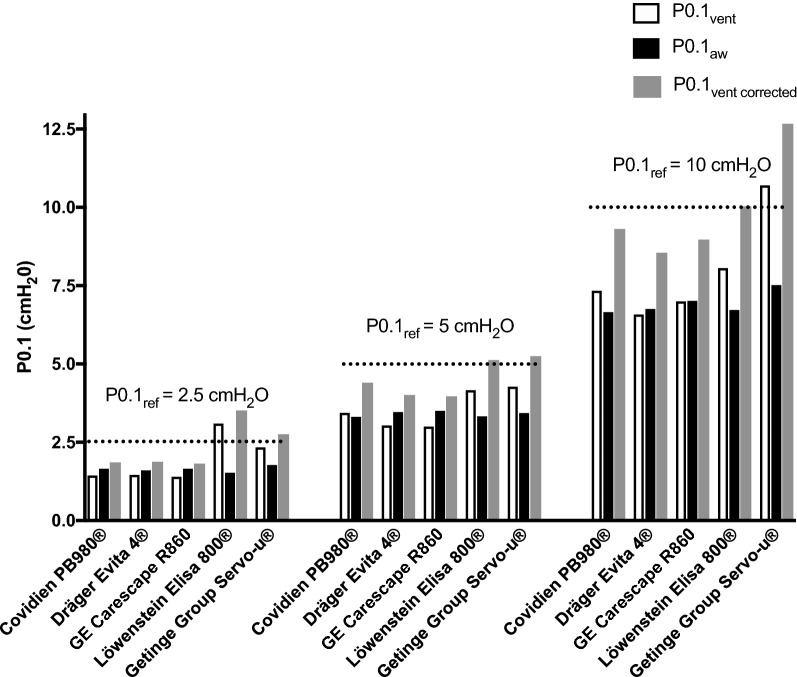
Automatically measured P0.1 displayed by the ventilator (P0.1_vent_), P0.1 measured on the airway pressure during automated P0.1 measurement (P0.1_aw_) and P0.1 measurement corrected for the occlusion location (P0.1_vent corrected_). P0.1_vent corrected_ = P0.1_vent_ + ∆P0.1_circuit_ with ∆P0.1_circuit_ corresponding to the difference between P0.1_circuit_ measured with an occlusion performed at 360 cm from the airway opening and P0.1_ref_ (reference P0.1) measured with an occlusion performed at the airway opening

Values of P0.1_vent_, P0.1_aw_ and P0.1_vent_ _corrected_ are presented for each inspiratory effort and each ventilator in Fig. 5.

## Discussion

This study shows that, for all the tested ventilators, relative changes in P0.1 values displayed on the ventilator screen correlate well with changes in reference P0.1. However, automated P0.1 measurements overall underestimate absolute P0.1 values with marked differences between the different ventilators. All of the tested ventilators perform an airway occlusion of at least 100 ms to measure P0.1, except one that surprisingly displays P0.1 values closer to the reference P0.1 than the others. Our data also suggest that ventilators’ inaccuracy to estimate P0.1 may be related to the location of the occlusion performed by the ventilator.

### Can technology explain the lack of accuracy of automated P0.1 measurements?

This study shows that different techniques are used by the different ventilators to measure P0.1. Most of the ventilators perform an airway occlusion of at least 100 ms as initially described. Interestingly, the Getinge Group Servo-u^®^ ventilator does not perform a specific occlusion to measure P0.1. The continuous P0.1 measurement during trigger delay (corresponding to an inspiratory occlusion) was described in the 90s when trigger delay was longer than 100 ms [9, 17]. As trigger delays were of about 40 ms in our model (in line with trigger delays measured in invasively mechanically ventilated patients with the new generation ventilators), the Getinge Group Servo-u^®^ ventilator probably extrapolates the slope of the airway pressure drop measured during the triggering phase to estimate P0.1. Of note, in our model, the values of P0.1_vent_ displayed by this ventilator were in general closer to the P0.1_ref_ values compared to the P0.1_vent_ displayed by the other tested ventilators. This suggests that the Getinge Group Servo-u^®^ ventilator may use an algorithm to accurately predict true P0.1.

Among the potential technological issues that may explain the lack of accuracy in P0.1 measurement, a non-complete occlusion of the inspiratory valve during the P0.1 measurement maneuver may reduce the airway pressure drop amplitude.

Interestingly, the good correlation between P0.1_vent_ and P0.1_aw_ suggests that the inaccuracy in pressure drop amplitude measurement performed by the ventilator cannot explain the documented inaccuracy.

Another explanation for P0.1_vent_ inaccuracy could be that, during automated P0.1 measurement, the airway occlusion is not performed by the ventilator at the airway opening but at the ventilator level, leading to a gas decompression in the circuit during the automated occlusion. The volume of gas trapped between the occlusion location and the airway opening depends on the circuit tubes’ length. The impact of the circuit length to explain inaccuracy in P0.1 measurement is corroborated by our results showing that the location of the occlusion impacts P0.1 measurement and that underestimation of P0.1 is higher when the circuit used is longer. The lack of concordance between our study and the study by Kulhen et al. [17] reporting a lower bias between P0.1 displayed by the Dräger Evita 1 ventilator and the reference P0.1 could potentially be explained by differences in the locations of the occlusion between the two models.

### Clinical implications

Overall, from a clinical point of view, the impact of this study is of importance. As previously said, in our model, the P0.1 measured values were lower than the reference values measured at the airway opening suggesting that new standard values should be determined for ventilated patients. The inaccuracy of P0.1 measurement was observed for each effort intensity. Of note, in absolute values, the differences between P0.1_vent_ and P0.1_ref_ were higher for higher respiratory drive. More importantly, for a given respiratory drive, P0.1 values displayed on the screen of various ventilators may be markedly different. Thus, it is not possible to define universal P0.1_vent_ thresholds for clinical purposes. This could explain why previous clinical studies in which P0.1 thresholds were used to predict extubation failure were not conclusive [18–20]. In addition, if a threshold value has to be defined, it should probably be defined for each given ventilator. From a general point of view, P0.1 variations may be of better interest than absolute values of P0.1. Thus, for a given patient, changes in P0.1 displayed by the ventilator are expected to reliably estimate changes in respiratory drive. This approach was used in some clinical studies to predict weaning outcome [10, 11, 19–22], to titrate positive end expiratory pressure in patients with dynamic hyperinflation [20] or to assess respiratory effort according to pressure support level [9, 21, 22].

Based on our results, a standardization of P0.1 measurement allowing to use unique absolute values of P0.1 in clinical practice, should at least require a standardization of the type, length and volume of the ventilator circuit and the performance of a complete occlusion of the inspiratory valve during P0.1 automated measurement maneuvers. Alternatively, the ventilator manufacturers could implement correction algorithms to adjust measured P0.1 values for these different factors.

### Study limitations

First, the main limitation of our study is that our results obtained with a lung model cannot be transposed to clinical settings without great caution. It must, however, be underlined that assessing different ventilators with different levels of inspiratory drive could not have been performed in patients. Importantly, it must be noted that one major difference between our model and patients is that our model expiratory volume is equal to 300 mL, which is much lower than end expiratory lung volume of ventilated patients (usually higher than 1 L). In addition, the shape of the simulated inspiratory effort may be different compared to the shape of the patient muscular pressure curve. However, the calibrated efforts used in the experiments were in the clinical range. A second major limitation of our study is that we tested only five ventilators. Other commercialized ventilators may have different performances. In addition, we only tested conventional servo-valve compressed-gas ventilators. Turbine-based ventilators may perform differently.

## Conclusion

In our model, agreement between P0.1 displayed on the ventilator screen (P0.1_vent_) and P0.1 measured on the airway pressure curve (P0.1_aw_) is good. Relative changes in P0.1 values displayed on the screen of all the tested ventilators correlate well with reference P0.1 changes. We can thus conclude that variations of P0.1_vent_ are reliable in all the tested ventilators. However, in our experimental conditions, accuracy of absolute values of P0.1_vent_ varies according to the ventilator model. P0.1_vent_ overall underestimates P0.1_ref_. Decompression of air in the circuit may partially explain some underestimation of P0.1_ref_ by the ventilators. This can potentially limit the use of universal P0.1 threshold values to make decisions in clinical practice.

## Supplementary information

**Additional file 1: Figure S1.** Airway pressure (Paw)–time curves illustrating P0.1_aw_ measurement in the ventilators performing an automatic occlusion of more than 100 ms to measure P0.1. Paw was measured with a pressure transducer inserted between the test lung and the Y piece of the circuit ventilator. P0.1_aw_ was defined by the Paw difference from the initial decrease in Paw to 100 ms after this initial decrease during the occlusion automatically performed by the ventilator for P0.1 measurement.

**Additional file 2: Figure S2.** Representative tracings of pressure and flow recordings during normal cycles (left) and during cycles with P0.1 automated measurements (right) in two tested ventilators for P0.1 reference of 2.5 cm H_2_O. A, Löwenstein Medical Elisa 800^®^; B, Getinge Group Servo-u^®^. Paw, airway pressure. Note that a short occlusion was performed in the Löwenstein Medical Elisa 800^®^ ventilator and that no occlusion was performed during automated P0.1 measurements in the Getinge Group Servo-u^®^ ventilator.

## Data Availability

The data sets analyzed during the current study are available from the corresponding author on reasonable request.

## Abbreviations

P0.1: Occlusion pressure at 100 ms
P0.1_ref_: Reference P0.1
P0.1_vent_: P0.1 automatically displayed on the ventilator screen
P0.1_aw_: P0.1 measured on the airway pressure–time curves
P0.1_circuit_: P0.1 values with occlusion performed at the end of circuit tubes of different lengths
∆P0.1_circuit_: Difference between P0.1_ref_ measured at the airway opening and P0.1 measured when the occlusion was performed at the end of a standard ventilator circuit
P0.1_vent corrected_: P0.1 values corrected for the effect of the location where the occlusion was performed in the circuit
