# Supplementary figures and images for: Accuracy of P0.1 measurements performed by ICU ventilators: a bench study
Source: Ann Intensive Care. 2019 Sep 13;9:104. doi: 10.1186/s13613-019-0576-x (PMC6744533; doi:10.1186/s13613-019-0576-x)

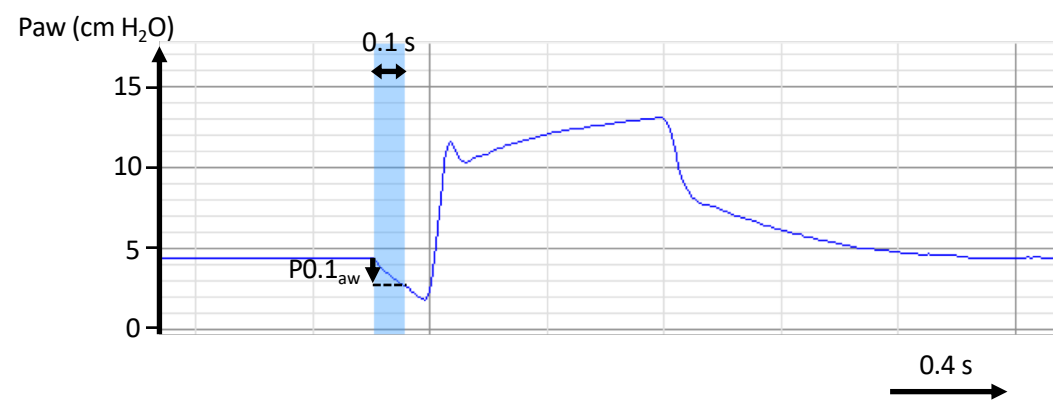

Supplement: Supplementary file 1 — Additional file 1: Figure S1. Airway pressure (Paw)–time curves illustrating P0.1aw measurement in the ventilators performing an automatic occlusion of more than 100 ms to measure P0.1. Paw was measured with a pressure transducer inserted between the test lung and the Y piece of the circuit ventilator. P0.1aw was defined by the Paw difference from the initial decrease in Paw to 100 ms after this initial decrease during the occlusion automatically performed by the ventilator for P0.1 measurement. [file 13613_2019_576_MOESM1_ESM.pdf]
