# Supplementary material for: Accuracy of P0.1 measurements performed by ICU ventilators: a bench study
Source: Ann Intensive Care. 2019 Sep 13;9:104. doi: 10.1186/s13613-019-0576-x (PMC6744533; doi:10.1186/s13613-019-0576-x)
Supplement: Supplementary file 2 — Additional file 2: Figure S2. Representative tracings of pressure and flow recordings during normal cycles (left) and during cycles with P0.1 automated measurements (right) in two tested ventilators for P0.1 reference of 2.5 cm H2O. A, Löwenstein Medical Elisa 800®; B, Getinge Group Servo-u®. Paw, airway pressure. Note that a short occlusion was performed in the Löwenstein Medical Elisa 800® ventilator and that no occlusion was performed during automated P0.1 measurements in the Getinge Group Servo-u® ventilator. [file 13613_2019_576_MOESM2_ESM.pdf]

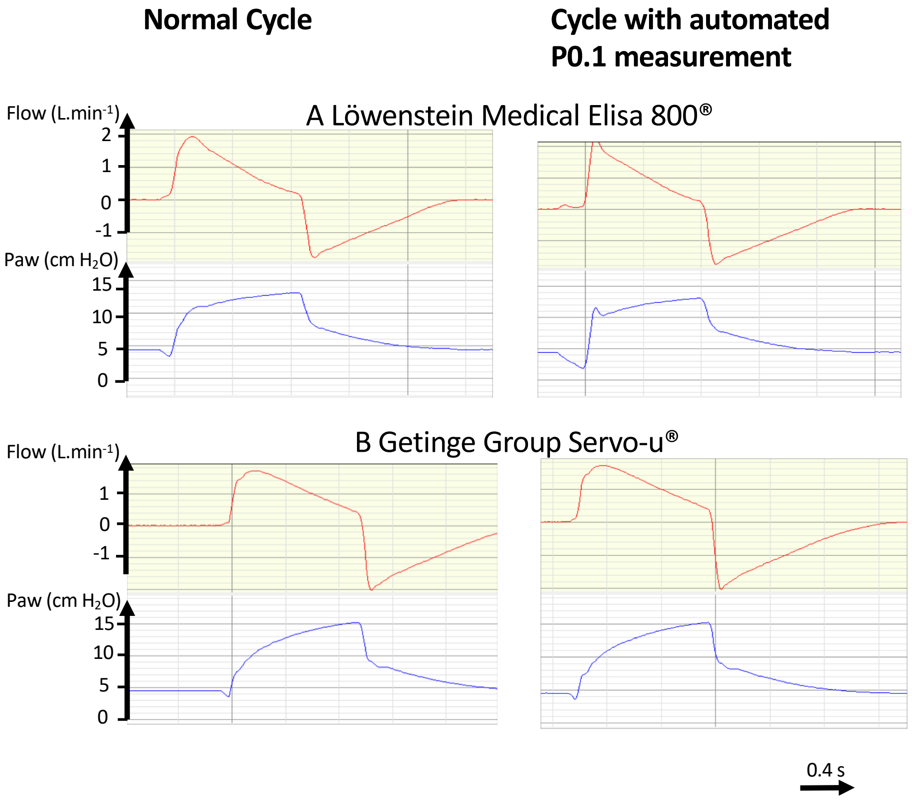

Normal Cycle
Cycle with automated P0.1 measurement
A Löwenstein Medical Elisa 800®
Flow (L.min-1)
2
1
0
-1
Paw (cm H2O)
15
10
5
0
B Getinge Group Servo-u®
Flow (L.min-1)
1
0
-1
Paw (cm H2O)
15
10
5
0
0.4 s
